# Why Are People High in Dispositional Awe Happier? The Roles of Meaning in Life and Materialism

**DOI:** 10.3389/fpsyg.2019.01208

**Published:** 2019-05-22

**Authors:** Huanhuan Zhao, Heyun Zhang, Yan Xu, Wen He, Jiamei Lu

**Affiliations:** ^1^Department of Psychology, Shanghai Normal University, Shanghai, China; ^2^School of Social Administration, Shanghai University of Political Science and Law, Shanghai, China; ^3^Beijing Key Laboratory of Applied Experimental Psychology, National Demonstration Center for Experimental Psychology Education (Beijing Normal University), Faculty of Psychology, Beijing Normal University, Beijing, China

**Keywords:** awe, dispositional awe, meaning in life, materialism, subjective well-being

## Abstract

Awe is an intense emotional response to perceptually vast stimuli that dramatically transcend one’s ordinary reference frame and provoke a need to adjust the current mental structures. Dispositional awe reflects individual differences in the tendency to experience awe. The current study aims to examine the effect of dispositional awe on subjective well-being, with a focus on confirming the mediating role of meaning in life and materialism. A sample of 563 Chinese adults completed measures of dispositional awe, meaning in life, materialism, and subjective well-being. Correlation analysis revealed that dispositional awe, meaning in life, and materialism were all significantly correlated with subjective well-being. Structural equation modeling showed significant paths from dispositional awe to subjective well-being through both meaning in life and materialism. Bootstrap analysis also indicated that meaning in life and materialism mediated the relationship between dispositional awe and subjective well-being. These findings not only corroborate the critical role of dispositional awe in promoting subjective well-being, but also shed some light on why people high in dispositional awe are happier than those low in dispositional awe. Limitations and directions for future research were also discussed.

## Introduction

Dispositional awe is an emotional disposition pertaining to people’s latent tendency to experience awe (Shiota et al., 2006). Awe is an intense emotional response to perceptually vast stimuli that dramatically transcend one’s ordinary reference frame and provoke a need to adjust the current mental structures (Keltner and Haidt, 2003; Shiota et al., 2007; Piff et al., 2015). Perceptual vastness and need for accommodation are the two core features of awe-eliciting stimuli (Keltner and Haidt, 2003; Shiota et al., 2007). The stimuli such as extraordinary nature scenes, glorious sunsets, impressive arts, charismatic leaders, intellectual epiphany, childbirth, religion, and spirituality can all inspire the strong emotional response of awe (Keltner and Haidt, 2003; Shiota et al., 2007; Saroglou et al., 2008; Zhao et al., 2018). Awe often includes feelings of wonder, amazement, enlightenment, elevation, admiration, and appreciation (Keltner and Haidt, 2003; Piff et al., 2015; Zhao et al., 2018). Although awe experiences may be tinged with fear and anxiety, awe is primarily considered a positive emotion (Gordon et al., 2016; Pearce et al., 2017; Stellar et al., 2017, 2018; Zhao et al., 2018).

From a personality perspective, dispositional awe reflects individual differences in the tendency to experience awe. It is a relatively permanent awe tendency, and the global pattern of individuals’ awe responses reflects their dispositional awe (Shiota et al., 2006). As a self-transcendent emotion, awe has been shown to enable people to transcend their daily concerns and mundane desires, diminish their emphasis on the individual self, shift their attention toward the needs of others, and encourage them to engage in various types of prosocial behaviors (Keltner and Haidt, 2003; Piff et al., 2015; Prade and Saroglou, 2016; Bai et al., 2017; Stellar et al., 2017, 2018; Jiang et al., 2018; Zhao et al., 2018). Awe plays a significant role in promoting the harmonious development of society. A thorough understanding of the socioemotional functioning of awe can thus provide us with valuable insights. Diener (2012) has maintained that abundant subjective well-being is an important aspect of a harmonious society. How to make people experience their lives in more positive ways has always been a focus of positive psychology researchers. Therefore, the main purpose of this study is to extend prior research by examining the effect of dispositional awe on subjective well-being and exploring the possible psychological mechanisms underlying this relationship.

### Dispositional Awe and Subjective Well-Being

Subjective well-being is sometimes labeled “happiness” (Diener, 2000). Such well-being is understood as people’s cognitive and affective evaluations of their lives as a whole and encompasses two most commonly studied components: life satisfaction and subjective happiness (Diener, 2000; Lyubomirsky, 2001; Diener et al., 2003; Szczygieł and Mikolajczak, 2017). Life satisfaction refers to one’s cognitive assessment of his life (Diener et al., 2003), whereas subjective happiness refers to one’s global assessment of whether he is a happy or an unhappy person (Lyubomirsky and Lepper, 1999). Hence, life satisfaction reflects a more cognitive facet of subjective well-being, whereas subjective happiness can be considered a rather affective facet of subjective well-being. Both life satisfaction and subjective happiness conceptualize well-being as a subjectively determined positive mental state, which represents the hedonic (versus eudaimonic) view of well-being (Ryan and Deci, 2001; Szczygieł and Mikolajczak, 2017).

Researchers on positive psychological have proposed that the self-transcendent emotion of awe may promote well-being or pleasure (Peterson and Seligman, 2004). A growing body of evidence has indicated that positive awe experience improves people’s subjective well-being (Rudd et al., 2012; Stellar et al., 2015; Gordon et al., 2016; Van Cappellen et al., 2016). For instance, using a sample of a nationwide panel, Rudd et al. (2012) found that awe inspired people’s love for life and enhanced their momentary life satisfaction by expanding their time perception. Gordon et al. (2016) revealed that participants who experienced positive awe in everyday life and in the laboratory reported higher levels of momentary well-being compared with those who did not experience awe. Moreover, the more frequently people reported feeling awe, the lower their levels of proinflammatory cytokines (Stellar et al., 2015). Some research also provided indirect evidence for predicting subjective well-being through dispositional awe. For example, Howell et al. (2013) found that nature setting and spirituality, as the major elicitors of awe, could enhance subjective well-being. Inspiration, which is central to the awe experience, exerts a positive influence on subjective well-being (Thrash et al., 2010). Therefore, as an emotional disposition, dispositional awe was assumed to promote subjective well-being.

Despite increasing evidence justifying the relationship between dispositional awe and subjective well-being, less attention has been paid to the psychological mechanisms underlying this relationship. Therefore, this research aims to complement earlier works by focusing on the roles of meaning in life and materialism as potential explanatory mechanisms for the association between dispositional awe and subjective well-being.

### Meaning in Life and Materialism as Potential Mechanisms

As a transcendental emotion, the experience of awe changes the way people view the world and gives them a new perspective on their lives (Algoe and Haidt, 2009; Schneider, 2009; Bonner and Friedman, 2011). Awe encourages people to focus more on their spiritual life and reduces the significance they attach to material pursuits (Rudd et al., 2012; Van Cappellen and Saroglou, 2012; Jiang et al., 2018). Self-determination theory suggests that intrinsic goal pursuit (e.g., meaning in life) contributes to subjective well-being, whereas extrinsic goal pursuit (e.g., materialism) does not (Ryan and Deci, 2000). A reasonable assumption within the framework of Fredrickson’s broaden-and-build theory (Fredrickson et al., 2008) is that dispositional awe might increase subjective well-being not only by broadening thinking and discovering the positive meaning in one’s life, but also by building enduring aspects of intrinsic aspirations and reducing one’s focus on extrinsic goals, such as materialism. Accordingly, in this study, we intended to test whether meaning in life and materialism would account for the relationship between dispositional awe and subjective well-being.

#### Meaning in Life as a Mediator

Meaning in life is defined as people’s beliefs that their lives are coherent, significant, and are endowed with a sense of overarching aim (Steger, 2009). It is a core facet of well-being and stems from an individual’s ability for self-transcendence (Cohen and Cairns, 2012; Howell et al., 2013). As stated, the subjective experience of awe is in accordance with the concept of self-transcendence (Shiota et al., 2014; Zhao et al., 2018). The awe experience makes people endorse spiritual beliefs (Saroglou et al., 2008; Van Cappellen and Saroglou, 2012), helps them ponder the meaning of life (Krause and Hayward, 2015), reassess their life goals and provides them a new understanding of the meaning of life (McDonald et al., 2009; Schneider, 2009; Bonner and Friedman, 2011). In support, positive emotions have been shown to increase people’s feeling that life has meaning (King et al., 2006). Based on these evidences, we proposed that dispositional awe would be positively associated with meaning in life.

Furthermore, a growing body of evidence has shown the important role of meaning in life in improving people’s subjective well-being. For instance, research has demonstrated that high meaning in life was related to life satisfaction (Steger et al., 2006; Ho et al., 2010) and positive affect (McMahan and Renken, 2011; Howell et al., 2013), whereas low meaning in life was closely related to psychological problems (Ho et al., 2010) and negative affect (McMahan and Renken, 2011). In addition, some indirect evidence confirmed that awe may enhance people’s sense of meaning in life (Thrash et al., 2010; Howell et al., 2013; Krause and Hayward, 2015), which ultimately promotes increased subjective well-being (Thrash et al., 2010; Howell et al., 2013). Thus, it is reasonable to hypothesize that meaning in life would be a potential mediator between dispositional awe and subjective well-being. Specifically, people high in dispositional awe would report higher meaning in life, which, in turn, would lead to higher subjective well-being.

#### Materialism as a Mediator

Another likely candidate to mediate the link between dispositional awe and subjective well-being is materialism, which refers to the elevated importance people attach to possessions and their acquisition as a necessary means to achieve the desired end states (Richins and Dawson, 1992). Materialists often regard material acquisition as the central focus of their lives, view material wealth as the main source of happiness and place great value on material possessions in judging one’s success (Richins and Dawson, 1992; Kashdan and Breen, 2007). Compared with non-materialists, highly materialistic people appear to be less globally satisfied with their lives and tend to experience more negative feelings (Burroughs and Rindfleisch, 2002; Kashdan and Breen, 2007; Dittmar et al., 2014; Tsang et al., 2014; Wang et al., 2017). Considerable research has suggested that materialism imposed a negative effect on subjective well-being. For example, a meta-analysis by Dittmar et al. (2014) demonstrated a consistent negative association between people’s materialistic pursuits in life and personal well-being. A recent longitudinal study on Chinese undergraduates shown that materialism decreased individuals’ subjective well-being and increased their depression (Wang et al., 2017). Reducing the level of materialism might be a critical step to enhance subjective well-being. As such, we expected that materialism would be negatively associated with subjective well-being.

On the other hand, previous studies have indicated that the transcendental nature of awe freed people from their worldly desires and dissuaded them from being preoccupied with materialistic concerns (Rudd et al., 2012; Jiang et al., 2018). For instance, Jiang et al. (2018) conducted two studies and discovered that experimentally inducing awe effectively weakened people’s desire for money. While awe reflected the values of self-transcendence (Shiota et al., 2014; Piff et al., 2015), materialism was located in the self-enhancement side of Schwartz’s model (Burroughs and Rindfleisch, 2002), rendering them opposite to each other. In particular, materialism preoccupied the individual with self and self-interest (Lambert et al., 2009), but awe diminished the importance of the individual self and self-interest (Piff et al., 2015; Zhao et al., 2018). Also, Weinstein et al. (2009) found that nature scenes, which are the major elicitors of awe experience, led to less materialistic aspirations. Therefore, it is logical to expect a negative link between dispositional awe and materialism. Taken together, we predicted that materialism may play a mediating role in the relationship between dispositional awe and subjective well-being. In other words, dispositional awe would possibly increase people’s subjective well-being by decreasing their materialism.

### Overview of the Present Study

**FIGURE 1:**
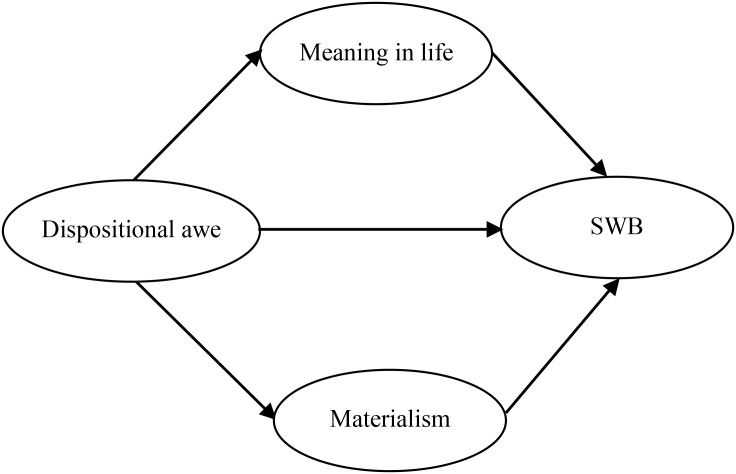
The hypothesized mediated model. SWB, subjective well-being.

The main purpose of this study was to extend previous research concerning the relationship between dispositional awe and subjective well-being and explore the potential mechanisms underlying this association. The hypotheses based on the aforementioned arguments and evidence are as follows: (1) dispositional awe significantly positively predicts subjective well-being; (2) meaning in life mediates the positive association between dispositional awe and subjective well-being; (3) materialism mediates the positive association between dispositional awe and subjective well-being. Specifically, people with higher dispositional awe reports higher meaning in life and lower materialism, which, in turn, result in higher levels of subjective well-being. The conceptual model of this study is shown in Figure 1.

## Materials and Methods

### Participants

A total of 600 Chinese adults were recruited from various organizations in China using Qualtrics survey software, of which 563 valid questionnaires were obtained for a 93.83% effective response rate. Participants were aged 29.14 (*SD* = 6.37) years on average, with an age range from 18 to 61 years. Three hundred and twenty-eight (58.26%) were females and 235 (41.74%) were males. Among the 563 participants, 104 (18.50%) of them had a high-school degree or below, 192 (34.10%) had a junior college degree, 243 (43.20%) had a bachelor degree, and 24 (4.30%) had a postgraduate degree. With regard to monthly income, 41 (7.30%) of the participants were less than 2000 yuan, 266 (47.20%) were between 2001 and 5000 yuan, 152 (27.00%) were between 5001 and 8000 yuan, 84 (14.90%) were between 8001 and 20000 yuan, and 20 (3.60%) were more than 20001 yuan.

### Measures

#### Dispositional Awe

Dispositional awe was assessed by the 38-item Dispositional Positive Emotion Scale (Shiota et al., 2006). This scale focuses on seven dispositional positive emotions: awe, joy, contentment, pride, love, compassion, and amusement. The subscale of dispositional awe includes six items (e.g., “I often feel awe”). Responses to items were rated on a seven-point Likert scale ranging from 1 (*strongly disagree*) to 7 (*strongly agree*), and higher scores indicated that individual has a higher level of dispositional awe. The Cronbach’s alpha of dispositional awe was 0.79.

#### Meaning in Life

The 10-item Meaning in Life Questionnaire was adopted to measure the participants’ meaning in life (Steger et al., 2006). Two dimensions of meaning in life are included: presence of meaning (e.g., I have discovered a satisfying life purpose) and search for meaning (e.g., I am always looking to find my life’s purpose). Each item was answered on a seven-point Likert scale ranging from 1 (*absolutely untrue*) to 7 (*absolutely true*), and higher scores reflected a higher level of meaning in life. The Cronbach’s alpha for the whole scale was 0.80, and 0.79 and 0.81 for presence of meaning and search for meaning.

#### Materialism

The 18-item Materialism Scale was used to assess the participants’ materialism value (Richins and Dawson, 1992). This scale consists of three dimensions: centrality (e.g., I like a lot of luxury in my life), happiness (e.g., I’d be happier if I could afford to buy more things), and success (e.g., “Some of the most important achievements in life includes acquiring material possessions”). Participants rated the items on a seven-point Likert scale ranging from 1 (*strongly disagree*) to 7 (*strongly agree*), and higher scores indicated a higher level of materialism. The Cronbach’s alpha for the whole scale was 0.82, and 0.72, 0.70, and 0.71 for centrality, happiness and success.

#### Subjective Well-Being

The subjective well-being scale includes two aspects of subjective well-being: a more cognition-based rating of life satisfaction was assessed by the Satisfaction with Life Scale (Diener et al., 1985) and a more affection-based rating of subjective happiness was assessed by the Subjective Happiness Scale (Lyubomirsky and Lepper, 1999; Szczygieł and Mikolajczak, 2017). The Satisfaction with Life Scale comprises five items (e.g., “I am satisfied with my life”), in which the participants were asked to indicate how much they agree or disagree with each item on a seven-point Likert scale ranging from 1 (*strongly disagree*) to 7 (*strongly agree*), and the corresponding Cronbach’s alpha was 0.87. The Subjective Happiness Scale includes four items (e.g., “Compared to most of my peers, I consider myself: less happy/more happy”), in which the participants were instructed to rate the first two item on a seven-point Likert scale ranging from 1 (*less happy*) to 7 (*more happy*), and the last two item from 1 (*not at all*) to 7 (*a great deal*). The corresponding Cronbach’s alpha was 0.85.

### Procedure

The present study was approved by the ethics board of the author’s institution. Research purposes and procedures were explained to the participants at the beginning and then signed an informed consent. Participants answered the series of self-reported questionnaires independently and anonymously within 25 min. Beyond the standard measures mentioned above, demographic information was also collected from each participant. After completing the whole survey, participants were thanked and debriefed.

### Data Analysis

First, following the procedure of Podsakoff et al. (2003), we employed Harman’s single-factor test to examine the common method bias. Then, we calculated the descriptive statistics and correlations among study variables by using SPSS 22.0.

Next, we adopted the two-step procedure introduced by Anderson and Gerbing (1988) to test the mediation effects. The measurement model was first analyzed to examine whether each of the four latent constructs was well represented by its indicators. To increase the reliability of parameter estimate, three item parcels were created as indicators of dispositional awe. The structural model was then analyzed using the maximum likelihood estimation in AMOS 21.0 program. In the structural equation modeling, the direct effect of dispositional awe on subjective well-being was first tested. Second, a mediated model containing mediators (meaning in life and materialism) and a direct path from dispositional awe to subjective well-being was tested. According to the suggestions by Hu and Bentler (1999), the model fit was generally considered acceptable when the RMSEA and SRMR values were below 0.08, and the GFI, IFI, and CFI values were above 0.90.

Finally, we performed the bootstrap analysis to calculate the bias-corrected percentile confidence intervals of the direct and indirect effects (MacKinnon, 2008) and further examine the mediation effects of meaning in life and materialism between dispositional awe and subjective well-being for significance in AMOS 21.0 (i.e., a bootstrap sample of 5000 was specified).

## Results

### Common Method Variance

Harman’s single-factor test was employed for statistically verifying the common method variance bias (Podsakoff et al., 2003). Exploratory factor analysis showed that nine factors were extracted and accounted for 59.20% of the variance among the items, and the first factor explained 19.05% of the variance. Moreover, Confirmatory factor analysis demonstrated that the single-factor model fit poorly with the data: χ^2^ (35, *N* = 563) = 547.35, χ^2^/*df* = 15.64, *p* < 0.001; RMSEA = 0.161; SRMR = 0.139; GFI = 0.82; IFI = 0.64; and CFI = 0.64). Hence, common method variance bias is not serious in the sample data.

### Preliminary Analyses

Table 1 presents the mean values, standard deviations and the bivariate correlations of the key variables measured in this study. In line with our expectations, dispositional awe was significantly and positively correlated with meaning in life (*r* = 0.37, *p* < 0.001) and subjective well-being (*r* = 0.38, *p* < 0.001), and negatively correlated with materialism (*r* = -0.18, *p* < 0.001). Thus, the hypothesis 1 was supported. Additionally, meaning in life was positively associated with subjective well-being (*r* = 0.37, *p* < 0.001), whereas materialism was negatively associated with subjective well-being (*r* = -0.31, *p* < 0.001).

**Table 1:** Mean values, standard deviations, and correlations for the key variables (*N* = 563).

	*M*	*SD*	1	2	3	4	5	6
(1) Dispositional awe	4.37	1.06	1					
(2) Meaning in life	4.75	0.88	0.37***	1				
(3) Materialism	3.79	0.78	–0.18***	–0.14***	1			
(4) Life satisfaction	3.78	1.41	0.33***	0.29***	–0.22***	1		
(5) Subjective happiness	4.61	1.22	0.33***	0.37***	–0.33***	0.52***	1	
(6) Subjective well-being	4.14	1.16	0.38***	0.37***	–0.31***	0.92***	0.82***	1


*^∗∗∗^*p* < 0.001.*

### Measurement Model

The full measurement model consisted of four latent constructs (i.e., dispositional awe, meaning in life, materialism, subjective well-being) and ten observed variables. The results demonstrated a satisfactory fit to the data: χ^2^ (29, *N* = 563) = 113.28, χ^2^/*df* = 3.91, *p* < 0.001; RMSEA = 0.072; SRMR = 0.062; GFI = 0.96; IFI = 0.94; and CFI = 0.94. All the standardized factor loadings for the indicators on the latent constructs were statistically significant (all *ps* < 0.001), suggesting that the four latent constructs were well operationalized by their respective indicators. Moreover, Skewness and kurtosis values for all variables were considered satisfactory (all < 1).

### Structural Model

**FIGURE 2:**
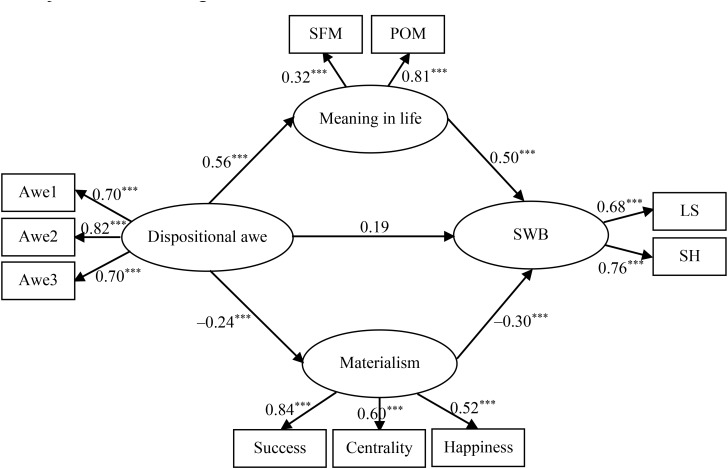
The mediated structural model. ^∗∗∗^*p* < 0.001; Awe1–Awe3, three parcels of dispositional awe; SFM, search for meaning; POM, presence of meaning; SWB, subjective well-being; LS, life satisfaction; SH, subjective happiness.

Structural equation modeling was adopted to analyze the mediation effect. The direct effect of dispositional awe on subjective well-being was first tested. Results showed that the direct standardized path (*β* = 0.54, *p* < 0.001) was significant. Then, a mediated model that contained the mediators (meaning in life and materialism) and a direct path from dispositional awe to subjective well-being was examined. Results indicated that the hypothesized meditational model fit the data well: χ^2^ (30, *N* = 563) = 119.40, χ^2^/*df* = 3.98, *p* < 0.001; RMSEA = 0.073; SRMR = 0.065; GFI = 0.96; IFI = 0.94; and CFI = 0.94. In addition, tests of parameter estimates revealed that all direct path coefficients were statistically significant in the hypothesized directions. The mediated model is shown in Figure 2. These results suggest that meaning in life and materialism play a mediating role in the relationship between dispositional awe and subjective well-being.

### Confidence Intervals of Direct and Indirect Effects

Table 2 displays the direct and indirect effects and their corresponding 95% confidence intervals. As demonstrated in Table 2, dispositional awe has a significant direct effect on subjective well-being. The indirect effects of dispositional awe on subjective well-being through meaning in life and materialism were also significant. Thus, Hypothesis 2 and 3, which state that meaning in life and materialism mediate the relationship between dispositional awe and subjective well-being, were supported.

**Table 2:** Standardized effects and 95% confidence intervals (CI).

Model pathways	Estimated effect	95% CI	
		Lower bonds	Upper bonds
**Direct effect**			
Dispositional awe → SWB	0.192	–0.127	0.386
Dispositional awe → Meaning in life	0.564^a^	0.380	0.733
Dispositional awe → Materialism	–0.238^a^	–0.361	–0.106
Meaning in life → SWB	0.498^a^	0.285	0.832
Materialism → SWB	–0.304^a^	–0.415	–0.191
**Indirect effect**			
Dispositional awe → (Meaning in life, Materialism) → SWB	0.353^a^	0.187	0.657


*^a^Empirical 95% confidence interval does not overlap with zero.*

## Discussion

This study explored the role of meaning in life and materialism as potential mechanisms for linking dispositional awe and subjective well-being. As predicted, dispositional awe was positively associated with both aspects of subjective well-being. More importantly, the structural equation modeling and bootstrap method further suggested that the effect of dispositional awe on subjective well-being was mediated by both meaning in life and materialism.

Our finding that dispositional awe was positively correlated with subjective well-being supported our first hypothesis. This result is in accordance with previous studies that explored the relationship between experimentally inducing awe and momentary well-being (Rudd et al., 2012; Gordon et al., 2016) and concurs with Fredrickson’s broaden-and-build theory, suggesting that positive emotions can significantly enhance individuals’ subjective well-being (Fredrickson et al., 2008). As a self-transcendent emotional disposition, a strong tendency to experience awe corresponds to a great sense of subjective well-being.

The main finding of this study – that both meaning in life and materialism mediates the link between dispositional awe and subjective well-being – confirmed our second and third hypothesis. Both mediation effect testing and bootstrap analysis provided powerful evidence that the paths from dispositional awe to subjective well-being through meaning in life or materialism were significant. The mediating role of meaning in life and materialism provides new insights into why people high in dispositional awe are happier.

On the one hand, dispositional awe was positively correlated with meaning in life. Research demonstrates that dispositional awe is imbued with a sense of meaning and fulfillment (Seaton and Beaumont, 2015), could open people’s mind, broaden their thinking, enable them to embrace new beliefs (Jiang et al., 2018), and increase the likelihood of experiencing positive meaning in their lives (Fredrickson and Joiner, 2002). Moreover, the finding that meaning in life is positively related to subjective well-being is in accordance with prior research (Steger et al., 2006; Ho et al., 2010; McMahan and Renken, 2011; Howell et al., 2013). Meaning in life has been identified as an important indicator of subjective well-being (Cohen and Cairns, 2012). Those who perceive their lives as high in meaning believed that their lives were significant, purposeful, and happy. Thus, individuals with high dispositional awe can interpret their life as more meaningful and have a more positive mental outlook, consequently making experiencing the happiness of life easier for them.

On the other hand, dispositional awe was negatively associated with materialism, which is consistent with the results of previous studies (Rudd et al., 2012; Van Cappellen and Saroglou, 2012; Jiang et al., 2018). People higher in dispositional awe paid less attention to mundane desires, attached less significance to materialistic concerns and had less inclination to pursue materialistic strivings. Conversely, materialistic striving seems to place a high priority on the self and on what one lacks and is trying to acquire (Lambert et al., 2009). Moreover, a significant negative association between materialism and subjective well-being was revealed in the current study. Low levels of materialism corresponds to high levels of subjective well-being experienced by people. This finding is also in line with prior research (Burroughs and Rindfleisch, 2002; Kashdan and Breen, 2007; Wang et al., 2017). Therefore, materialism has a mediating effect in the relationship between dispositional awe and subjective well-being.

In summary, data from this study demonstrated the influence of dispositional awe on subjective well-being through the mediating effects of meaning in life and materialism. Findings suggest that people high in dispositional awe appeared to gain subjective well-being due to their high tendency to experience meaning in life and low tendency to hold a materialistic belief. Meaning in life and materialism were two important ways to affect subjective well-being, and a high tendency to experience awe encouraged people to find the positive meaning in life and discouraged them from striving for material wealth excessively. This study improves our understanding of the effect of dispositional awe on subjective well-being.

### Limitations and Future Directions

Several limitations of the current study warrant further investigation. First, a cross-sectional, correlational design was adopted, which means caution should be applied when interpreting the causal direction of the associations. Experimental or longitudinal studies in future research would further increase the reliability of the conclusions. Second, this study relied on self-reported measures exclusively. Multiple measures (e.g., peers, parents, or teachers reports) might be used in future surveys to ensure the objectivity of the results. Third, this study was limited to hedonic well-being. Future research could examine whether the same pattern of the findings holds when eudaimonic well-being is considered (Ryan and Deci, 2001). Fourth, only Chinese adults were included in the study sample, which may reduce the generalizability of our findings to other cultures. Future research should be conducted in more diverse cultures to establish the universality of our results.

### Implications

Despite the abovementioned limitations, this research was the first attempt to directly and simultaneously explore meaning in life and materialism as explanatory mechanisms for the relationship between dispositional awe and subjective well-being. People high in dispositional awe attain a high sense of subjective well-being mainly because of their high level of meaning in life and low level of materialism. From a theoretical perspective, these findings expand the existing literature and may offer us a valuable foundation for future research on the socioemotional functioning of dispositional awe. From a practical perspective, this work provides us with valuable implications for how to promote human well-being. The positive association between dispositional awe and subjective well-being indicates that fostering people’s dispositional awe and evoking their awe experiences may contribute to improve subjective well-being. In addition to traveling, for instance, traveling to Tibet (Van Cappellen and Saroglou, 2012) or coastal wilderness (Pearce et al., 2017), cultivating the personality trait of absorption (van Elk et al., 2016), improving the ability to appreciate beauty and excellence (Guüsewell and Ruch, 2012), and practicing the loving-kindness meditation (Stell and Farsides, 2016) can all help nurturing awe in individuals. Moreover, the mediating role of meaning in life and materialism in the relationship between dispositional awe and subjective well-being suggests that directing interventions aimed at increasing meaning in life (e.g., achieving higher self-concept clarity; Shin et al., 2016) and weakening materialism (e.g., reflecting on intrinsic values; Lekes et al., 2012) may also be relatively effective strategies to enhance people’s subjective well-being.

## Data Availability

The datasets generated for this study are available on request to the corresponding author.

## Ethics Statement

The present study was approved by the research ethics committee of Shanghai Normal University. Research purposes and procedures were explained to the participants at the beginning and then signed an informed consent.

## Author Contributions

HuZ designed the study and wrote the manuscript. HuZ and HeZ acquired and analyzed the data. HeZ, YX, WH, and JL provided instruction and advice for the study. All authors approved the final version of the manuscript.

## Conflict of Interest Statement

The authors declare that the research was conducted in the absence of any commercial or financial relationships that could be construed as a potential conflict of interest.

## References

[B1] Algoe S. B. Haidt J. (2009). Witnessing excellence in action: the “other-praising” emotions of elevation, gratitude, and admiration. *J. Posit. Psychol.* 4 105–127. 10.1080/17439760802650519 19495425 PMC2689844

[B2] Anderson J. C. Gerbing D. W. (1988). Structural equation modeling in practice: a review and recommended two-step approach. *Psychol. Bull.* 103 411–423. 10.1037//0033-2909.103.3.411

[B3] Bai Y. Maruskin L. A. Chen S. Gordon A. M. Stellar J. E. McNeil G. D. (2017). Awe, the diminished self, and collective engagement: universals and cultural variations in the small self. *J. Pers. Soc. Psychol.* 113 185–209. 10.1037/pspa0000087 28481617

[B4] Bonner E. T. Friedman H. L. (2011). A conceptual clarification of the experience of awe: an interpretative phenomenological analysis. *Humanist. Psychol.* 39 222–235. 10.1080/08873267.2011.593372

[B5] Burroughs J. E. Rindfleisch A. (2002). Materialism and well-being: a conflicting values perspective. *J. Consumer Res.* 29 348–370. 10.1086/344429

[B6] Cohen K. Cairns D. (2012). Is searching for meaning in life associated with reduced subjective well-being? Confirmation and possible moderators. *J. Happiness Stud.* 13 313–331. 10.1007/s10902-011-9265-7

[B7] Diener E. (2000). Subjective well-being: the science of happiness and a proposal for a national index. *Am. Psychol.* 55 34–43. 10.1037//0003-066X.55.1.3 11392863

[B8] Diener E. (2012). New findings and future directions for subjective well-being research. *Am. Psychol.* 67 590–597. 10.1037/a0029541 23163434

[B9] Diener E. Emmons R. A. Larsen R. J. Griffin S. (1985). The satisfaction with life scale. *J. Pers. Assess.* 49 71–75. 10.1207/s15327752jpa4901_13 16367493

[B10] Diener E. Oishi S. Lucas R. E. (2003). Personality, culture, and subjective well-being: emotional and cognitive evaluations of life. *Annu. Rev. Psychol.* 54 403–425. 10.1146/annurev.psych.54.101601.145056 12172000

[B11] Dittmar H. Bond R. Hurst M. Kasser T. (2014). The relationship between materialism and personal well-being: a meta-analysis. *J. Pers. Soc. Psychol.* 107 879–924. 10.1037/a0037409 25347131

[B12] Fredrickson B. L. Cohn M. A. Coffey K. A. Pek J. Finkel S. M. (2008). Open hearts build lives: positive emotions, induced through loving-kindness meditation, build consequential personal resources. *J. Pers. Soc. Psychol.* 95 1045–1062. 10.1037/a0013262 18954193 PMC3156028

[B13] Fredrickson B. L. Joiner T. (2002). Positive emotions trigger upward spirals toward emotional well-being. *Psychol. Sci.* 13 172–175. 10.1111/1467-9280.00431 11934003

[B14] Gordon A. M. Stellar J. E. Anderson C. L. McNeil G. D. Loew D. Keltner D. (2016). The dark side of the sublime: distinguishing a threat-based variant of awe. *J. Pers. Soc. Psychol.* 113 310–328. 10.1037/pspp0000120 27929301

[B15] Guüsewell A. Ruch W. (2012). Are there multiple channels through which we connect with beauty and excellence? *J. Posit. Psychol.* 7 516–529. 10.1080/17439760.2012.726636

[B16] Ho M. Y. Cheung F. M. Cheung S. F. (2010). The role of meaning in life and optimism in promoting well-being. *Pers. Individ. Dif.* 48 658–663. 10.1016/j.paid.2010.01.008 23144651

[B17] Howell A. J. Passmore H.-A. Buro K. (2013). Meaning in nature: meaning in life as a mediator of the relationship between nature connectedness and well-being. *J. Happiness Stud.* 14 1681–1696. 10.1007/s10902-012-9403-x

[B18] Hu L. T. Bentler P. M. (1999). Cutoff criteria for fit indexes in covariance structure analysis: conventional criteria versus new alternatives. *Struct. Equ. Modeling* 6 1–55. 10.1080/10705519909540118

[B19] Jiang L. Yin J. Mei D. Zhu H. Zhou X. (2018). Awe weakens the desire for money. *J. Pac. Rim Psychol.* 12:e4 10.1017/prp.2017.27

[B20] Kashdan T. B. Breen W. L. (2007). Materialism and diminished well-being: experiential avoidance as a mediating mechanism. *J. Soc. Clin. Psychol.* 26 521–539. 10.1521/jscp.2007.26.5.521

[B21] Keltner D. Haidt J. (2003). Approaching awe, a moral, spiritual, and aesthetic emotion. *Cogn. Emot.* 17 297–314. 10.1080/02699930244000318 29715721

[B22] King L. A. Hicks J. A. Krull J. L. Del Gaiso A. K. (2006). Positive affect and the experience of meaning in life. *J. Pers. Soc. Psychol.* 90 179–196. 10.1037/0022-3514.90.1.179 16448317

[B23] Krause N. Hayward R. D. (2015). Awe of God, congregational embeddedness, and religious meaning in life. *Rev. Relig. Res.* 57 219–238. 10.1007/s13644-014-0195-9

[B24] Lambert N. M. Fincham F. D. Stillman T. F. Dean L. R. (2009). More gratitude, less materialism: the mediating role of life satisfaction. *J. Posit. Psychol.* 4 32–42. 10.1080/17439760802216311

[B25] Lekes N. Hope N. H. Gouveia L. Koestner R. Philippe F. L. (2012). Influencing value priorities and increasing well-being: the effects of reflecting on intrinsic values. *J. Posit. Psychol.* 7 249–261. 10.1080/17439760.2012.677468

[B26] Lyubomirsky S. (2001). Why are some people happier than others? The role of cognitive and motivational processes in well-being. *Am. Psychol.* 56 239–249. 10.1037/0003-066X.56.3.239 11315250

[B27] Lyubomirsky S. Lepper H. S. (1999). A measure of subjective happiness: preliminary reliability and construct validation. *Soc. Indic. Res.* 46 137–155. 10.1023/A:1006824100041

[B28] MacKinnon D. P. (2008). *Introduction to Statistical Mediation Analysis.* Mahwah: NJ: Erlbaum.

[B29] McDonald M. G. Wearing S. Ponting J. (2009). The nature of peak experience in wilderness. *Humanist. Psychol.* 37 370–385. 10.1080/08873260701828912

[B30] McMahan E. A. Renken M. D. (2011). Eudaimonic conceptions of well-being, meaning in life, and self-reported well-being: initial test of a mediational model. *Pers. Individ. Dif.* 51 589–594. 10.1016/j.paid.2011.05.020

[B31] Pearce J. Strickland-Munro J. Moore S. A. (2017). What fosters awe-inspiring experiences in nature-based tourism destinations? *J. Sustain. Tour.* 25 362–378. 10.1080/09669582.2016.1213270

[B32] Peterson C. Seligman M. (2004). *Character Strengthsand Virtues: A Handbook and Classification.* New York, NY: Oxford University Press.

[B33] Piff P. K. Dietze P. Feinberg M. Stancato D. M. Keltner D. (2015). Awe, the small self, and prosocial behavior. *J. Pers. Soc. Psychol.* 108 883–899. 10.1037/pspi0000018 25984788

[B34] Podsakoff P. M. MacKenzie S. B. Lee J. Y. Podsakoff N. P. (2003). Common method biases in behavioral research: a critical review of the literature and recommended remedies. *J. Appl. Psychol.* 88 879–903. 10.1037/0021-9010.88.5.879 14516251

[B35] Prade C. Saroglou V. (2016). Awe’s effects on generosity and helping. *J. Posit. Psychol.* 11 522–530. 10.1080/17439760.2015.1127992

[B36] Richins M. L. Dawson S. (1992). A consumer values orientation for materialism and its measurement: scale development and validation. *J. Consumer Res.* 19 303–316. 10.1086/209304

[B37] Rudd M. Vohs K. D. Aaker J. (2012). Awe expands people’s perception of time, alters decision making, and enhances well-being. *Psychol. Sci.* 23 1130–1136. 10.1177/0956797612438731 22886132

[B38] Ryan R. M. Deci E. L. (2000). Self-determination theory and the facilitation of intrinsic motivation, social development, and well-being. *Am.Psychol.* 55 68–78. 10.1037/0003-066X.55.1.68 11392867

[B39] Ryan R. M. Deci E. L. (2001). On happiness and human potentials: a review of research on hedonic and eudaimonic well-being. *Annu. Rev. Psychol.* 52 141–166. 10.1146/annurev.psych.52.1.141 11148302

[B40] Saroglou V. Buxant C. Tilquin J. (2008). Positive emotions as leading to religion and spirituality. *J. Posit. Psychol.* 3 165–173. 10.1080/17439760801998737

[B41] Schneider K. J. (2009). *Awakening to Awe: Personal Stories of Profound Transformation.* Lanham, MD: Jason Aronson.

[B42] Seaton C. L. Beaumont S. L. (2015). Pursuing the good life: a short-term follow-up study of the role of positive/negative emotions and ego-resilience in personal goal striving and eudaimonic well-being. *Motiv. Emot.* 39 813–826. 10.1007/s11031-015-9493-y

[B43] Shin J. Y. Steger M. F. Henry K. L. (2016). Self-concept clarity’s role in meaning in life among american college students: a latent growth approach. *Self Identity* 15 206–223. 10.1080/15298868.2015.1111844

[B44] Shiota M. N. Keltner D. John O. P. (2006). Positive emotion dispositions differentially associated with big five personality and attachment style. *J. Posit. Psychol.* 1 61–71. 10.1080/17439760500510833

[B45] Shiota M. N. Keltner D. Mossman A. (2007). The nature of awe: elicitors, appraisals, and effects on self-concept. *Cogn. Emot.* 21 944–963. 10.1080/02699930600923668

[B46] Shiota M. N. Thrash T. M. Danvers A. F. Dombrowski J. T. (2014). “Transcending the self: awe, elevation, and inspiration,” in *Handbook of Positive Emotions*, eds Tugade M. Shiota M. Kirby L. (New York, NY: Guilford Press), 362–377.

[B47] Steger M. F. (2009). “Meaning in life,” in *Oxford Handbook of Positive Psychology*, eds Snyder C. R. Lopez S. J. (New York: NY: Oxford University Press), 679–687.

[B48] Steger M. F. Frazier P. Oishi S. Kaler M. (2006). The meaning in life questionnaire: assessing the presence of and search for meaning in life. *J. Couns. Psychol.* 53 80–93. 10.1037/0022-0167.53.1.80 23406365

[B49] Stell A. J. Farsides T. (2016). Brief loving-kindness meditation reduces racial bias, mediated by positive other-regarding emotions. *Motiv. Emot.* 40 140–147. 10.1007/s11031-015-9514-x

[B50] Stellar J. E. Gordon A. Anderson C. L. Piff P. K. McNeil G. D. Keltner D. (2018). Awe and humility. *J. Pers. Soc. Psychol.* 114 258–269. 10.1037/pspi0000109 28857578

[B51] Stellar J. E. Gordon A. Piff P. K. Anderson C. L. Cordaro D. Bai Y. (2017). Self-transcendent emotions and their social functions: compassion, gratitude, and awe bind us to others through prosociality. *Emot. Rev.* 9 200–207. 10.1177/1754073916684557

[B52] Stellar J. E. John-Henderson N. Anderson C. L. Gordon A. M. McNeil G. D. Keltner D. (2015). Positive affect and markers of inflammation: discrete positive emotions predict lower levels of inflammatory cytokines. *Emotion* 15 129–133. 10.1037/emo0000033 25603133

[B53] Szczygieł D. Mikolajczak M. (2017). Why are people high in emotional intelligence happier? They make the most of their positive emotions. *Pers. Individ. Dif.* 117 177–181. 10.1016/j.paid.2017.05.051

[B54] Thrash T. M. Elliot A. J. Maruskin L. A. Cassidy S. E. (2010). Inspiration and the promotion of well-being: tests of causality and mediation. *J. Pers. Soc. Psychol.* 98 488–506. 10.1037/a0017906 20175626

[B55] Tsang J.-A. Carpenter T. P. Roberts J. A. Frisch M. B. Carlisle R. D. (2014). Why are materialists less happy? The role of gratitude and need satisfaction in the relationship between materialism and life satisfaction. *Pers. Individ. Dif.* 64 62–66. 10.1016/j.paid.2014.02.009

[B56] Van Cappellen P. Saroglou V. (2012). Awe activates religious and spiritual feelings and behavioral intentions. *Psychol. Relig. Spiritual.* 4 223–236. 10.1037/a0025986

[B57] Van Cappellen P. Toth-Gauthier M. Saroglou V. Fredrickson B. L. (2016). Religion and well-being: the mediating role of positive emotions. *J. Happiness Stud.* 17 485–505. 10.1007/s10902-014-9605-5

[B58] van Elk M. Karinen A. Specker E. Stamkou E. Baas M. (2016). Standing in awe: the effects of awe on body perception and the relation with absorption. *Collabra* 2:4 10.1525/collabra.36

[B59] Wang R. Liu H. Jiang J. Song Y. (2017). Will materialism lead to happiness? A longitudinal analysis of the mediating role of psychological needs satisfaction. *Pers. Individ. Dif.* 105 312–317. 10.1016/j.paid.2016.10.014

[B60] Weinstein N. Przybylski A. K. Ryan R. M. (2009). Can nature make us more caring? Effects of immersion in nature on intrinsic aspirations and generosity. *Pers. Soc. Psychol. Bull.* 35 1315–1329. 10.1177/0146167209341649 19657048

[B61] Zhao H. Zhang H. Xu Y. Lu J. He W. (2018). Relation between awe and environmentalism: the role of social dominance orientation. *Front. Psychol.* 9:2367. 10.3389/fpsyg.2018.02367 30559692 PMC6286991

